# Recent Development of Optoelectronic Application Based on Metal Halide Perovskite Nanocrystals

**DOI:** 10.3389/fchem.2021.822106

**Published:** 2022-01-05

**Authors:** Jianxiu Hao, Xing Xiao

**Affiliations:** ^1^ School of Chemistry and Chemical Engineering, Suzhou University, Suzhou, China; ^2^ Department of Orthopedic Surgery, The First Affiliated Hospital of Shandong First Medical University, Jinan, China

**Keywords:** metal halide perovskite, nanocrystals, solar cells, light-emitting diodes, lasers

## Abstract

In the past years, metal halide perovskite (MHP) single crystals have become promising candidates for optoelectronic devices since they possess better optical and charge transport properties than their polycrystalline counterparts. Despite these advantages, traditional bulk growth methods do not lend MHP single crystals to device integration as readily as their polycrystalline analogues. Perovskite nanocrystals (NCs), nanometer-scale perovskite single crystals capped with surfactant molecules and dispersed in non-polar solution, are widely investigated in solar cells and light-emitting diodes (LEDs), because of the direct bandgap, tunable bandgaps, long charge diffusion length, and high carrier mobility, as well as solution-processed film fabrication and convenient substrate integration. In this review, we summarize recent developments in the optoelectronic application of perovskite nanocrystal, including solar cells, LEDs, and lasers. We highlight strategies for optimizing the device performance. This review aims to guide the future design of perovskite nanocrystals for various optoelectronic applications.

## Introduction

Metal halide perovskite materials have drawn great attention for optoelectronic applications due to their superior electrical properties (Chen et al., 2017; Zeng et al., 2018). They are a class of materials with a formula of ABX_3_ in which A is a monovalent cation, B is a divalent metal ion, and X is a halide anion (Chen et al., 2019a). The outstanding optical and electrical properties of metal halide perovskites include high absorption coefficient, high carrier mobility, long carrier lifetime, long carrier diffusion length, and high defect tolerance (Wang et al., 2017). After only 10-year development, the efficiency of perovskite solar cells has rocketed from 3.8% to 25.5%, showing great potential for commercial application (Feng et al., 2020). Besides, metal halide perovskite materials are also widely investigated in other optoelectronic devices, such as sensitive photodetectors and x-ray detectors, lasers, and light-emitting diodes (LEDs) (Chen et al., 2019b; Sun et al., 2020).

Polycrystalline thin films and single crystals are two forms of metal halide perovskite materials for optoelectronic application. The former contains large amounts of grain boundaries that are rich in charge traps, causing adverse effect on the optoelectronic properties, and stability of the perovskite materials (Lin et al., 2018; Zheng et al., 2019). In comparison, perovskite single crystals are free of grain boundaries and are demonstrated with lower defect density, better optoelectronic properties, and higher stability than the polycrystalline thin films (Jiang et al., 2020a). Dong et al. observed the ultra-low trap state density of 10^10^ cm^−3^ and ultra-long charge carrier diffusion lengths of 175 μm under one Sun illumination in methylammonium lead iodide (MAPbI_3_) single crystals (Dong et al., 2015). Chen et al. reported that mixed cation and mixed halide perovskite single crystals remained stable even after 10,000 h water-oxygen and 1,000 h light aging (Chen et al., 2019a). In fact, it is universally recognized that perovskite single crystals are intriguing for higher-performance and more stable optoelectronic devices (Cheng et al., 2020).

The morphology control is a key factor determining the optoelectronic application of perovskite single crystals (Bao et al., 2017). Millimeter- or centimeter-sized perovskite bulk single crystals are ideal candidates for high-energy radiation detection due to the large thickness and existence of heavy atom (Wu et al., 2021). However, their large thickness leads to ineffective carrier collection and thus low external quantum efficiency (EQE) of solar cells. Besides, the challenges of integration with substrates and low photoluminescence quantum yield (PLQY) also limits application of bulk single crystals (Jiang et al., 2019). In this case, single crystal thin films (SCTF), micro single crystals, single crystal wire/plate, and single crystal quantum dots are developed for high-performance solar cells, photodetectors, lasers, and LEDs, respectively (Shao et al., 2017). For example, the efficiency of single crystal solar cells reaches 21.1% when using SCTF with a thickness of 20 um, which is competitive with the perovskite polycrystalline solar cells (Chen et al., 2019c).

Perovskite nanocrystals (NCs), nanometer-scale perovskite single crystals capped with surfactant molecules and dispersed in non-polar solution (Wang et al., 2021), are promising for optoelectronic applications, such as solar cells, LEDs, lasers, scintillation (Wang et al., 2019), and solar concentrators (Liu et al., 2021), due to their convenient deposition on conductive substrates based on solution-based processes (Zhao et al., 2019a; Zeng et al., 2019). Significant research efforts have been achieved for passivating defects in perovskite NCs, pushing the performance of perovskite NC-based optoelectronic devices better and better. In this manuscript, we summarize progress in perovskite NC solar cells, light-emitting diodes, and lasers, as well as challenges and possible solutions.

## Perovskite NC Solar Cells

The large crystal thickness hinders application of perovskite bulk single crystals in photovoltaic application. Recently, space-confined strategy has been widely used to grow perovskite single-crystal thin films and efficient solar cells are achieved (Chen et al., 2019c). In this method, the lateral size of the single-crystal thin films is only several millimeters, leading to small-sized solar cells. In contrast, perovskite NCs can be processed by spin-coating, blade-coating to achieve large-area devices, which can satisfy the requirement of commercial application.

**FIGURE 1:**
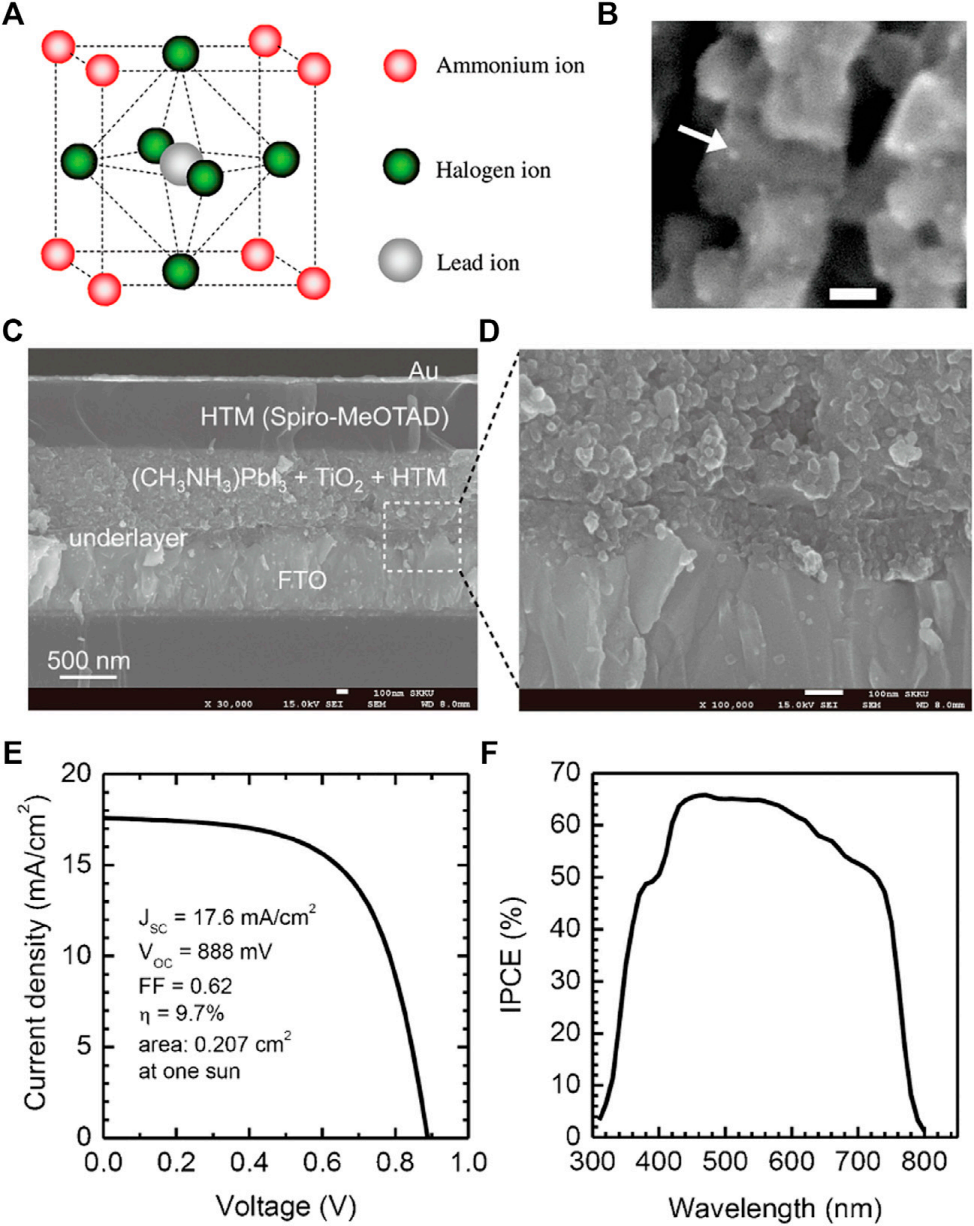
**(A)** Crystal structures of perovskite compounds. **(B)** SEM image of particles of nanocrystalline CH_3_NH_3_PbBr_3_ deposited on the TiO_2_ surface. The arrow indicates a particle, and the scale bar shows 10 nm. Reproduced with permission (Wang et al., 2019). Copyright 2009, American Chemical Society. **(C)** Cross-sectional SEM image of the device. **(D)** Active layer–underlayer–FTO interfacial junction structure. **(E)** Photocurrent density as a function of the forward bias voltage. **(F)** EQE as a function of incident wavelength. Reproduced with permission (Zeng et al., 2019). Copyright 2012, Springer Nature.

The first perovskite NC solar cells belong to the “dye-sensitized” type, and the MAPbI_3_ and MAPbBr_3_ NCs were employed as the sensitizer (Figure 1A). The NCs were prepared using a templated-based approach, with 2–3 nm in diameter (Figure 1B), yielding a PCE of 3.8% (Kojima et al., 2009). Several years later, the device performance increased to 6.54% through surface modification of the TiO_2_ electron transport layer (ETL) and post-anneal of the devices (Im et al., 2011). Later, the liquid electrolyte was replaced with a solid hole transport layer (HTL), the spiro-OMeTAD film, to enhance the device stability (Figures 1C,D). Sensitized meso-TiO_2_ devices showing PCEs of 8% and 9.7% (Figures 1E,F) were achieved using MAPbI_3_ and MAPbI_2_Cl NCs, respectively (Kim et al., 2012). Through replacing the meso-TiO_2_ with meso-Al_2_O_3_, the device PCE further improved to 10.9% (Lee et al., 2012). The Al_2_O_3_ is a large bandgap semiconductor, which is inefficient for electron extraction (Figure 1C), revealing that the perovskites can also act as a charge-transporting material (Sum and Mathews, 2014). To better control over the perovskite NC growth over mesoporous metal oxide, a sequential deposition method was introduced by Gratzel and coworkers (Burschka et al., 2013). Limited PbI_2_ NCs around 22 nm were deposited over a nanoporous TiO_2_ at the first step (Figure 1D), then transformed into perovskite NCs through exposing to a MAI solution (Figure 1E). This method offers much controllable device morphology than previously reported and led to a much improved PCE of 15% (Figure 1F).

**FIGURE 2:**
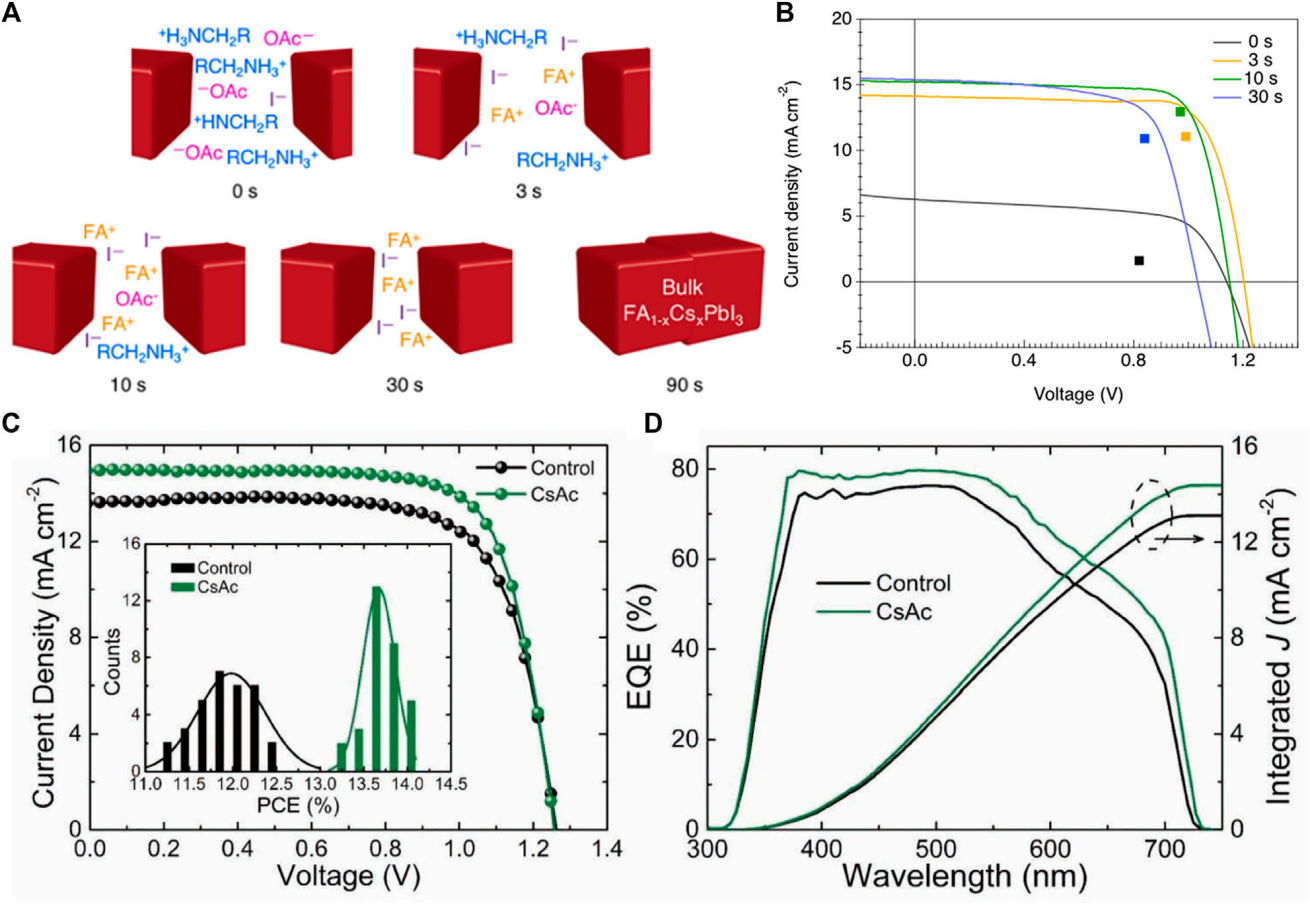
**(A)** A cartoon of surface composition showing CsPbI_3_ films that were treated with MeOAc and then with solutions of FAI in EtOAc for 3, 10, 30, and 90 s. **(B)** Current density–voltage with stabilized power output shown as square markers. Reproduced with permission (Sum and Mathews, 2014). Copyright 2018, American Chemical Society. **(C)** J–V curves of the best cells without (control) and with CsAc post-treatment and PCE distribution histograms (inserted) of 32 control and CsAc-treated cells, measured under reverse scan. **(D)** EQE curves and integrated J of the best cells. Reproduced with permission (Burschka et al., 2013). Copyright 2019, Wiley-VCH.

The size distribution and crystal surface of template-synthesized perovskite NCs, which is the basis of device performance, are difficult to control. To overcome this, solution-chemistry synthesis methods were employed to produce high-quality perovskite NCs through ligand control. Luther et al. synthesized 9-nm α-CsPbI_3_ NCs using the hot-injection method (Swarnkar et al., 2016), and purified the NCs using methyl acetate (MeOAc) as an antisolvent to remove surface ligands without inducing agglomeration or defect states. A planer device structure was employed. The best-performing device, employing CsPbI_3_ NCs with an E_g_ of 1.73 eV, showed 10.77% PCE for a device made and tested under ambient conditions. The maximum device V_OC_ has reached ∼85% of the NC bandgap, but the J_SC_ is limited, which mainly suffers from the high electric resistance due to the presence of capping ligands. In order to overcome this limitation, Luther and coworkers post-treated the NC film using a cation halide (AX) salt, which has greatly enhanced the charge carrier mobility (Sanehira et al., 2017). With the help of FAI post-treatments, the device J_SC_ was greatly enhanced; thus, a high PCE of 13.4% was achieved. Luther’s group went further into the FAI treatment chemistry by time-of-flight secondary ion mass spectrometry in a subsequent study (Wheeler et al., 2018). They demonstrated that initial FAI treatments lead to strong coupling across the thickness of the film, but there also exists a concentration gradient that transforms the film into a FA-rich bulk phase by extended treatment time (Figures 2A,B), providing basic rules for fabrication of high-quality, electronically coupled perovskite NC films that maintain quantum confinement. After that, Ma and coworkers developed a cesium acetate post-treatment method for CsPbI_3_ NCs to fill the NC surface vacancy and improve electron coupling between NCs. As a result, the carrier lifetime, diffusion length, and mobility of the CsPbI_3_ NC film were improved, delivering an impressive efficiency of 14.01% for CsPbI_3_ NC cells (Figures 2C,D) (Ling et al., 2019). The cesium acetate-treated CsPbI_3_ NC devices exhibit improved stability against moisture due to the improved NC surface environment. Very recently, Luther and coworkers demonstrated that optimizing the heterojunction position as well as the composition will greatly change the device performance, and they have successfully enhanced the PCE of CsPbI_3_ NC solar cells to a high value of 17.39% by introducing the charge separating heterostructure (Zhao et al., 2019b).

**FIGURE 3:**
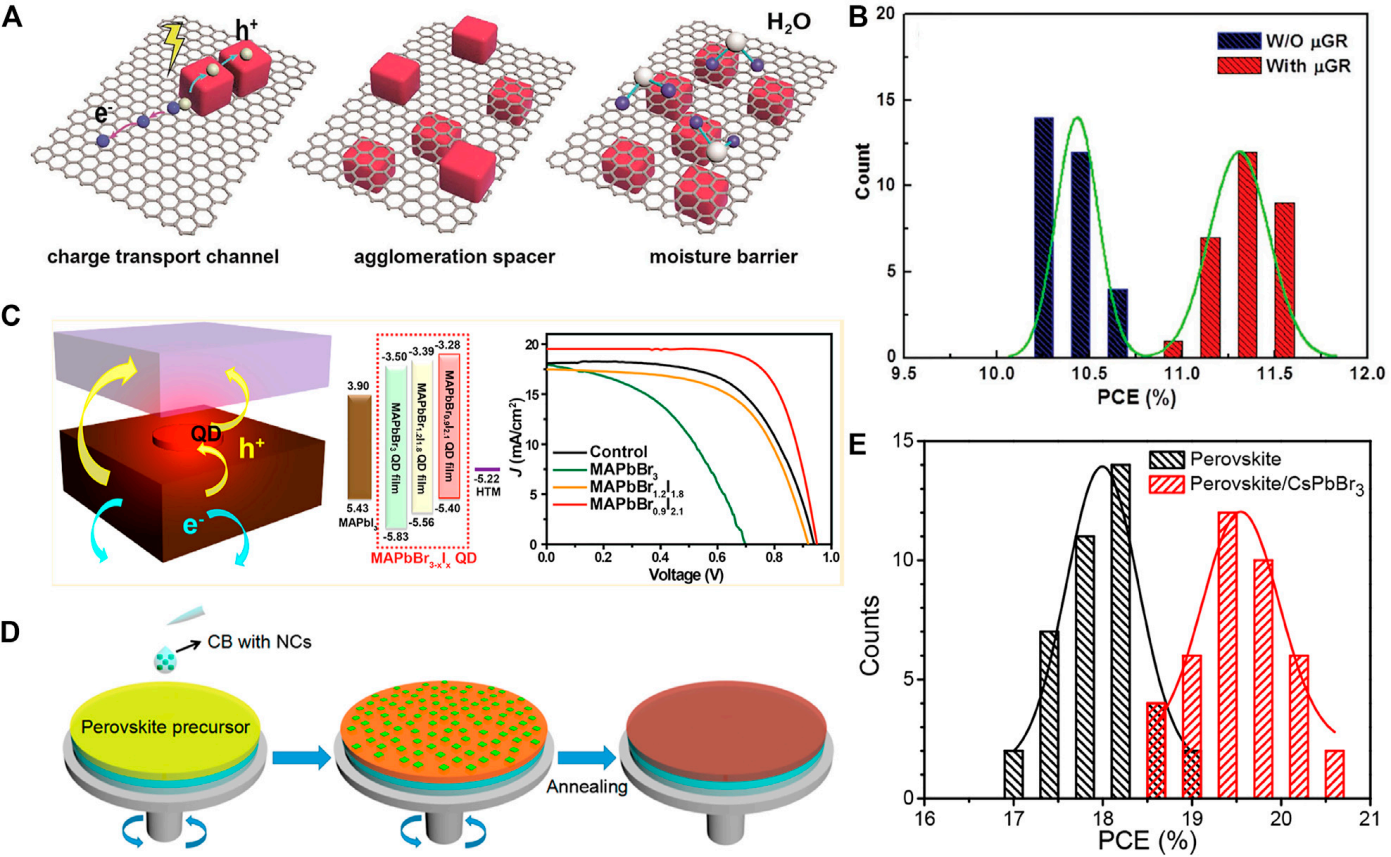
**(A)** Schematic drawing of the charge transport process and stabilization mechanism for the µGR/CsPbI_3_ NC-based solar cells. **(B)** Comparison of the PCE distribution for the CsPbI_3_ and µGR/CsPbI_3_ devices. Reproduced with permission (Sanehira et al., 2017). Copyright 2018, Wiley-VCH. **(C)** Schematic illustration, Energy diagram, and J–V curves of devices employing MAPb(BrI)_3_ NCs as the interface-regulating material. Reproduced with permission (Yuan et al., 2018). Copyright 2016, American Chemical Society. **(D)** Schematic diagram of the deposition method for the perovskite film with CsPbBr_3_ NCs; the green dots represent CsPbBr_3_ NCs. **(E)** Histogram of solar cell efficiencies for 40 devices fabricated without and with 2 mg/ml CsPbBr_3_ NCs in the dripping solvent. Reproduced with permission (Chen et al., 2015a). Copyright 2018, American Chemical Society.

At the same time, other strategies have also been developed to improve the electronic coupling. The charge carrier transport properties of CsPbI_3_ NC films were greatly enhanced by using μ-graphene to cross-link CsPbI_3_ NCs (Wang et al., 2018), increasing the device PCE to 11.4%, together with enhanced moisture and thermal stabilities (Figures 3A,B). Yuan et al. employed a dopant-free polymeric (PTB7) as the hole transport materials, realizing a loss energy loss (0.45 V) and thus a high PCE of 12.55% (Yuan et al., 2018). Liu and coworkers prepared highly stable CsPbI_3_ NCs with the assistance of GeI_2_ and achieved stable devices (85% retained of the initial performance after storage for 90 days) with a PCE of 12.15% (Liu et al., 2019). There are also attempts to commercialize; for that, CsPbBr_3_ NC inks have been prepared through replacing the bulky organic ligands by short low-boiling-point ligands during synthesis (Akkerman et al., 2016). Films from the CsPbBr_3_ NC inks showed high conductivity, which is benefiting from the short capping ligands, and the corresponding cells exhibited a decent PCE of 5.4% and good stability.

Benefiting from the easily tuned bandgap, the perovskite NCs have been used as interface materials to optimize the interfacial band alignment of solution-processed solar cells (Chen et al., 2015a). For example, MAPb(BrI)_3_ NCs have been chosen to engineer the interface between MAPbI_3_ films and HTLs. The energy levels of MAPb(BrI)_3_ NCs were adjusted by changing the Br:I ratios, and with the help of MAPbBr_0.9_I_2.1_ NC films, the device performance has been enhanced by 29% (Figure 3C), suggesting that the hole extraction was improved (Cha et al., 2016). All inorganic perovskite NCs with better thermal stability have also been employed as interface materials (Zhang et al., 2018a). For example, Bian and co-workers used an assembled film with CsPbI_3_ NCs to optimize the energy-level alignment for better carrier collection. The authors developed multiple strategies including Mn^2+^ doping, FAI treatment, and thiocyanate capping to the perovskite NCs, resulting in reduced trap states, enhanced carrier mobility, and improved chemical stability, and thus higher device PCE (Bian et al., 2018). Recently, Zai et al. used CsPbBr_3_ NC solution as the antisolvent to prepare FAMAPb(I_0.85_Br_0.15_)_3_ films, resulting in reduced carrier recombination process and more favorable energy alignment, which boosted the device PCE to 20.56% (Figures 3D,E) (Zai et al., 2018). We summarized the component of perovskite NCs employed and corresponded device structures and performances in Table 1.

**TABLE 1:** Overview of some representative references for perovskite NCs-based photovoltaics.

Component	Device structure	PCE (%)	Year	Ref.
α-CsPbI_3_	FTO/TiO_2_/CsPbI_3_ NCs/spiro-OMeTAD/MoO_x_/Al	10.77	2016	25
δ-CsPbBr_3_	FTO/TiO_2_/CsPbBr_3_ NCs/spiro-OMeTAD/Au	5.4	2016	33
α-CsPbI_3_	FTO/TiO_2_/CsPbI_3_ NCs/spiro-OMeTAD/MoO_x_/Al	13.43	2017	26
α-CsPbI_3_	FTO/TiO_2_/CsPbI_3_ NCs/PTB7/MoO_x_/Ag	12.55	2018	31
α-CsPbI_3_	FTO/TiO_2_/CsPbI_3_ NCs/spiro-OMeTAD/Au	12.15	2019	32
α-CsPbI_3_	FTO/TiO_2_/CsPbI_3_ NCs/PTAA/MoO_x_/Ag	14.1	2019	28
α-CsPbI_3_	ITO/TiO_2_/Cs_0.25_FA_0.75_PbI_3_/CsPbI_3_/spiro-OMeTAD/MoO_x_/Al	17.39	2019	29
MAPbBr_3-x_I_x_	FTO/TiO_2_/MAPbI_3_ film/MAPbBr_0.9_I_2.1_ NCs/spiro-OMeTAD/Cr/Au	13.32	2016	35
CsPbBrI_2_	FTO/TiO_2_/CsPbBrI_2_ film/CsPbBrI_2_ NCs/PTAA/Au	14.12	2018	36
CsPbI_3_	FTO/TiO_2_/CsPbBrI_2_ film/CsPbI_3_ NCs/PTAA/Au	14.45	2018	37
CsPbBr_3_	ITO/SnO_2_/FA_0.85_MA_0.15_Pb(Br_0.15_I_0.85_)_3_ film mixed with CsPbBr_3_ NCs/spiro-OMeTAD/Au	20.56	2018	38

Since charge carrier transport, which is determined by the carrier mobilities and carrier lifetime, determines the solar cell performance (Chen et al., 2013a), it is crucial to prepare high-quality active layers with low trap density towards high PCEs (Yao et al., 2015). Being different from the bulk perovskites, charge carrier transport mechanisms in perovskite NC films include resonant energy transfer, variable range hopping, and tunneling between adjacent NCs (Chen et al., 2015b). Carrier transport through all these mechanisms can be enhanced by reducing the inter-nanocrystal distance, which means that exchanging long original ligands to short ligands or removing the surface ligands will lead to high-quality perovskite NC films with excellent carrier transport performance. To date, the perovskite NC film thicknesses in best-performing cells are approximately 200 nm, which is thinner than that (∼500 nm) in bulk film devices, indicating that the photocurrent of NC cells can become higher through increasing the active layer thickness while ensuring efficient carrier transport. Thus, efforts should be paid on developing new methods towards efficient ligand exchange and ligand removing. Besides, beyond the single-junction photovoltaics, combining perovskite NC cells with other type of devices to form tandem solar cells may also be promising.

## Perovskite NC LEDs

**FIGURE 4:**
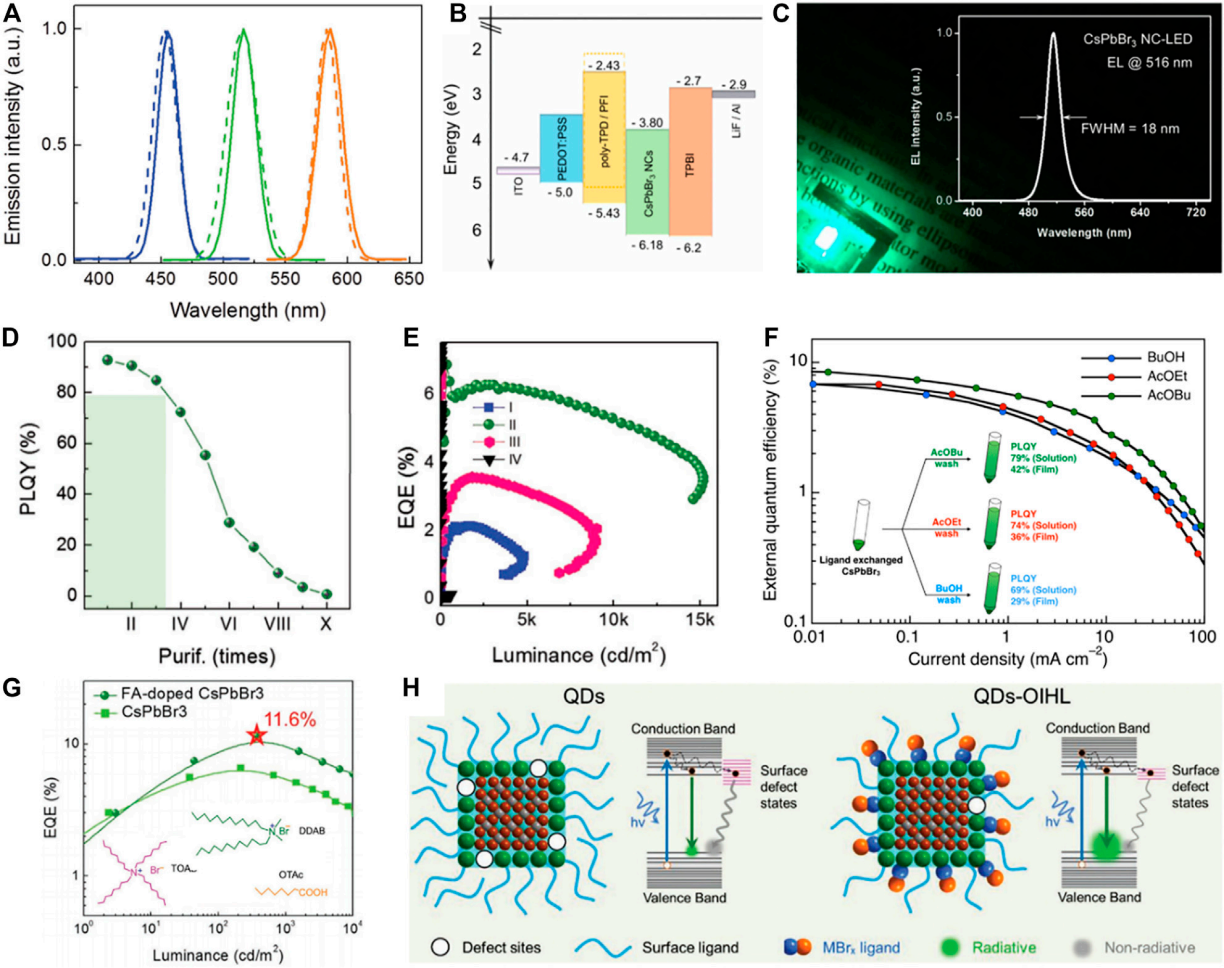
**(A)** The EL spectra (straight line) of red-, green-, and blue-perovskite NC LEDs, and the PL spectra (dashed line) of NCs dispersed in hexane. Reproduced with permission (Zai et al., 2018). Copyright 2018, Wiley-VCH. **(B)** Overall energy band diagram of the LED structure. The hole injection becomes more efficient after introducing the PFI. **(C)** A photo of a bright CsPbBr_3_ NC LED, with its narrow EL spectrum as inset. Reproduced with permission (Chen et al., 2013a). Copyright 2018, American Chemical Society. **(D)** PL QY of CsPbBr_3_ NC inks with different purifying cycles dispersed in hexane. **(E)** EQE as a function of luminance of devices with different purifying cycles. Reproduced with permission (Song et al., 2015). Copyright 2018, Wiley-VCH. **(F)** EQE as a function of current density of devices employing NCs that use different washing process with BuOH, AcOEt, and AcOBu, respectively. Reproduced with permission (Zhang et al., 2016a). Copyright 2018, American Chemical Society. **(G)** EQE of the devices as a function of luminance. Inset shows the surface ligands employed. Reproduced with permission (Sutherland and Sargent, 2016). Copyright 2018, Wiley-VCH. **(H)** Schematic description of radiative and nonradiative recombination of NCs. Reproduced with permission (Zhang et al., 2016b). Copyright 2018, Wiley-VCH.

Room-temperature electroluminescence (EL) of perovskite NC LEDs was first demonstrated by Prieto and coworkers in 2014. Free MAPbBr_3_ NCs with a quantum yield (QY) of 20% were used as the emitters, and only low brightness and poor device performance were reported. The brightness of perovskite NC-based LEDs was improved by Zeng and coworkers by employing bright CsPbBr_3_ NCs (PL QY over 85%) as the emitters, which reaches 946 cd m^−2^ under the voltage of 8.8 V. Blue and red LEDs with mixed halogen component perovskite NCs have also been shown (Figure 4A) (Song et al., 2015). Later, Zhang et al. enhanced the hole injection efficiency by introducing a thin film of perfluorinated ionomer (PFI) sandwiched between the hole transporting layer and the CsPbBr_3_ emitting layer (Figure 4B), which led to a narrow EL emission at 516 nm with FWHM = 18 nm and a peak brightness of 1,377 cd m^−2^ (Figure 4C) (Zhang et al., 2016a). Those initial works highlighting the promise of perovskite NCs for applications in light emission (Sutherland and Sargent, 2016).

Then, the performances of CsPbBr_3_ NC LEDs entered a rapid development stage. Through using dual-phase CsPbBr_3_-CsPb_2_Br_5_ composites, Sun and coworkers reduced exciton diffusion length and decreased the trap density of perovskite NC films, which led to an enhanced EQE of 2.21% (Zhang et al., 2016b). Later, Zeng and coworkers developed a ligand density control method to balance the surface passivation and carrier injection (Figure 4D), and an EQE of 6.27% was obtained for the CsPbBr_3_ LEDs (Figure 4E) (Li et al., 2017a). Kido and coworkers employed a similar method, that is, to wash the CsPbBr_3_ NCs several times with butyl acetate, to remove excess ligands from the NCs (Chiba et al., 2017). Their NC-LED exhibited a maximum EQE of 8.73% (Figure 4F), revealing the important role of the NC surface towards high device performance. The EQE of CsPbBr_3_ NC-LEDs increased to 11.6% through FA cation doping and employing a group of short surface ligands including TOAB (tetraoctylammonium bromide), DDAB (didodecyldimethylammonium bromide), and OTAc (octanoic acid) (Figure 4G) (Song et al., 2018a). To further passivate perovskite NCs and improve electrical transportation properties of NC films, Zeng and coworkers developed a general organic–inorganic hybrid ligand strategy (Figure 4H), which has led to a maximum peak EQE of 16.48%, the highest value for green NC-based LEDs to date (Song et al., 2018b).

**FIGURE 5:**
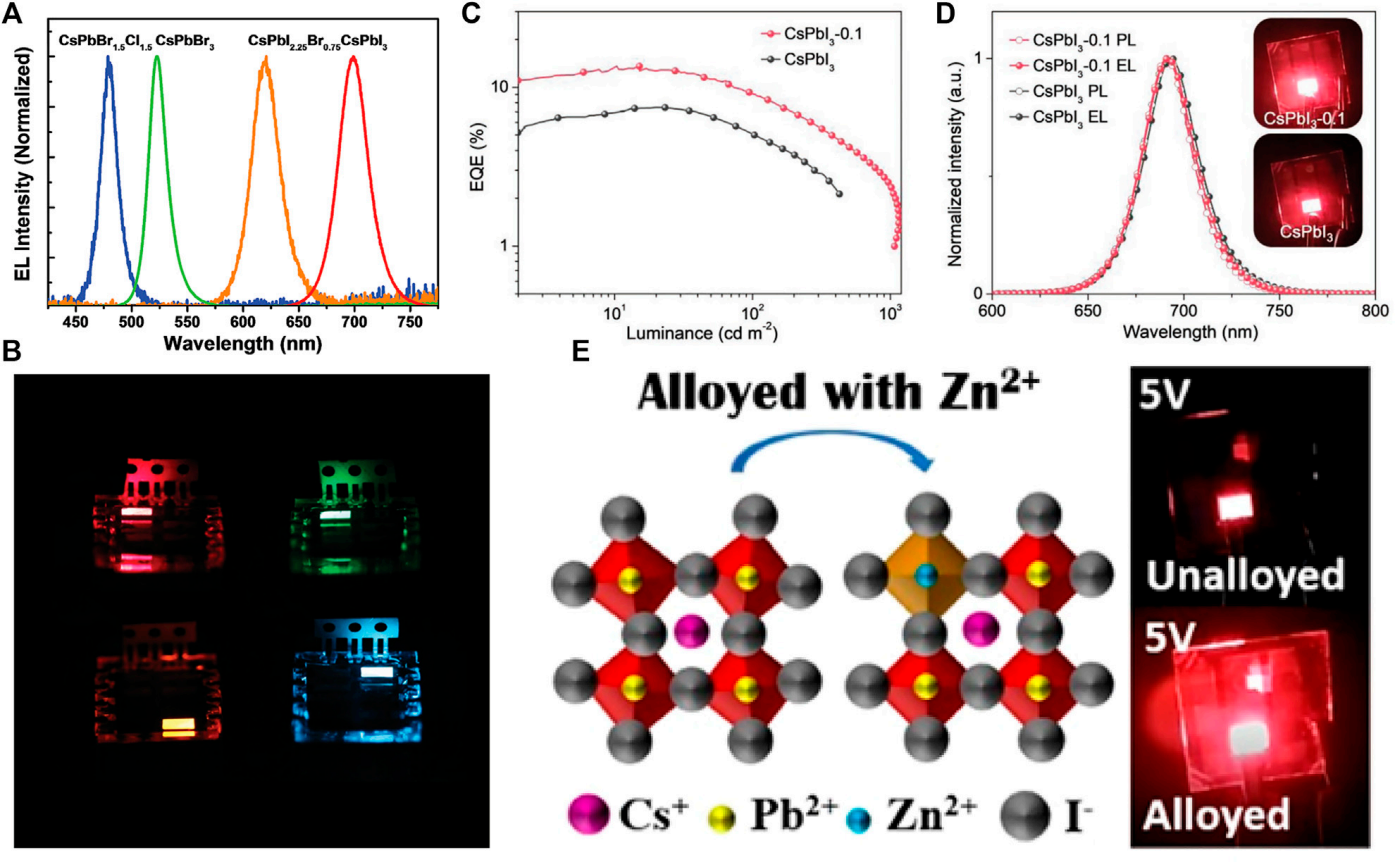
**(A)** Electroluminescence spectra of red-, orange-, green-, and blue-emitting perovskite nanocrystal LEDs. **(B)** Images of perovskite nanocrystal LEDs in operation. Reproduced with permission (Li et al., 2017a). Copyright 2016, Wiley-VCH. **(C)** External quantum efficiency versus luminance of the same devices. **(D)** PL and EL spectra of the CsPbI_3_ and CsPbI_3_-0.1 NC-based LED; inset shows photographs of the two devices. Reproduced with permission (Li et al., 2016). Copyright 2016, American Chemical Society. **(E)** Schematic diagram of the Zn^2+^ doping for the CsPbI_3_ NCs and the light from the respective working devices. Reproduced with permission (Zhang et al., 2016c). Copyright 2018, American Chemical Society.

Compared to NC LEDs based on the bromine components, efficient iodine-based perovskite NC LEDs are more difficult to obtain because of the unstable nature of the iodine-based NC materials. Initial studies of CsPbI_3_ NC LEDs were focused on their device performances. Through a trimethylkaluminum (TMA) vapor-based cross-linking method, the electron-hole capture ability of the compact CsPbI_3_ NC film was enhanced, giving rise to high-performance red LEDs with a peak EQE of 5.7% (Figures 5A,B) (Li et al., 2016). Later, Zhang et al. demonstrated that through a simple post treatment to the CsPbI_3_ NCs with polyethylenimine (PEI), the NC surface defects could be well passivated, leading to a remarkable EL efficiency of 7.25% (Zhang et al., 2016c). After that, the researchers began to think over the device stability as well. Pan et al. passivated CsPbI_3_ NCs using a bidentate ligand 2,2′-iminodibenzoic acid (IDA), and obtained bright NCs with improved stability. Although the performance of IDA passivated LEDs was lower than previously reported values, the corresponded device stability was enhanced (Pan et al., 2018). Soon after that, both the performance and the stability of CsPbI_3_ NC LEDs were greatly enhanced by using PbS to cap the CsPbI_3_ NCs. Zhang et al. developed a strategy to simultaneously enhance the optical properties and stability of CsPbI_3_ NCs without damaging the semiconducting properties, which is realized by epitaxial growth of PbS semiconductor on the surface of CsPbI_3_ NCs. With PbS capping, the CsPbI_3_ NC film switched from *n*-type behavior to nearly ambipolar, allowing to fabricate LEDs using *p-i-n* structures. The thus-fabricated LEDs showed enhanced storage and operation stability, and an EQE of 11.8% (Zhang et al., 2018b). To make the LEDs more stable and efficient, Lu et al. developed a method to improve both the PL and the EL efficiency through using SrCl_2_ as a co-precursor when synthesizing CsPbI_3_ NCs (Figures 5C,D). As a result, NCs with simultaneous Sr doping and Cl surface passivation were obtained, and devices using these emitters showed enhanced stability and a high EQE of 13.5% (Lu et al., 2018). Soon after that, the device EQE was further improved to 15.1% through using Zn-alloyed CsPbI_3_ NCs as the emitters (Figure 5E) (Shen et al., 2019). The most efficient and stable NCs have the component of CsPb_0.64_Zn_0.36_I_3_.

Except for NC LEDs based on those pure Br or I component NCs, devices that employ mixed halogen perovskite NCs have also been studied. Mixed halogen NCs offer more choices on the emitting color, which is promising in high-purity color display. We summarized some representative results of perovskite NC LEDs in Table 2. To date, the most efficient perovskite NC LEDs that exhibited an EQE of 21.3% are based on CsPb(Br/I)_3_ NCs, which is obtained through anion exchange between CsPbBr_3_ NCs and ammonium iodine salts (Chiba et al., 2018).

**TABLE 2:** Overview of some representative references for perovskite NCs-based LEDs.

Material	EQE_max_ (*%*)	CE_max_ (*cd/A*)	L_max_ (*cd/m* ^*2*^)	V_on_ (*V*)	Year	Ref.
CsPbBr_3_	8.73		1,660	2.6	2017	47
CsPbBr_3_	11.6	45.5	55,800	≈2.4	2018	48
CsPbI_3_	11.8	0.81	1,050	1.9	2018	53
CsPb(Br/I)_3_	21.3		794	2.7	2018	56
CsPbI_3_	13.5		1,152	2	2018	54
CsPbBr_3_	16.48	66.7	76,940	2.4	2018	49
CsPb_0.64_Zn_0_._36_I_3_	15.1		2,202	2.0	2019	55

### Perovskite NC Lasers

Lasers are devices that can emit light through an optical amplification process, which takes advantage of the stimulated emission of electromagnetic radiation (Yakunin et al., 2015). Perovskite NCs offer bright tunable emission and are flexibly afforded by colloidal synthesis, ensuring that they are promising for laser applications. Sun and coworkers employed CsPbX_3_ NCs (PL from 470 to 620 nm) with sizes of approximately 10 nm to fabricate thin films and demonstrated room-temperature amplification of spontaneous emission in the visible spectral range. The PL peak position changed with pump intensities, and the PL spectra become narrower (FWHM = 5 nm) when the pump intensity was increased (Wang et al., 2015). The threshold was reduced to as low as 5 μJ cm^−2^ (400 nm at 100 fs) by using whispering-gallery-mode (WGM) lasing in which CsPbX_3_ NCs were coated onto silica spheres. The laser possessed high modal net gain values of at least 450 ± 30 cm^−161^. Xu et al. demonstrated that CsPbBr_3_ NCs can excite large optical gain (>500 cm^−1^) in thin films, and the clear stable two-photon pumped lasing for CsPbBr_3_ NCs doped in microtubule resonators has a threshold of 0.8 mJ cm^−2^ (Xu et al., 2016). Li et al. fabricated bright perovskite NC-SiO_2_ composite films by anchoring NCs onto silica nanospheres, which show random lasing with thresholds down to 40 μJ cm^−2^ (400 nm at 100 fs) (Li et al., 2017b). These examples reveal the strong nonlinear properties in the emerging perovskite NCs and suggest that CsPbX_3_ lasers hold promise for future nonlinear photonic devices.

## Challenges and Perspective

The past years have witnessed great development of optoelectronic devices based on perovskite NCs; however, the most recent development is relatively sluggish. To provide instructive guidelines for future development, the challenges in this research field are discussed and possible solutions are proposed.

1) The PLQY of perovskite NCs films is usually smaller than solution, a general problem for any kind of NCs (Zhou et al., 2011), which should be increased to improve the EQE of LEDs. The decrease of PLQY may be due to the aggregation of perovskite NCs in solid state. To overcome this problem, construction of core-shell structures may be promising towards highly emissive solid-state perovskite NCs (Chen et al., 2014).
2) The electronic coupling between adjacent NCs are vital for carrier transport for both NCs solar cells and LEDs (Chen et al., 2013b). The existence of long-chain surfactant can passivate the surface dangling bond, but is adverse for carrier transport. Therefore, developing advanced ligand exchange strategy is required to ensure effective carrier transport and defect passivation. Learning from PbS NCs solar cells, bidentate ligand containing N, S atoms can interact with adjacent NCs and may solve this key challenges.
3) The toxic lead ions of halide perovskite materials are harmful for researchers and environment. Lots of advanced encapsulation techniques have been developed to avoid leakage of lead ions. In comparison, developing lead-free perovskite materials can overcome this problem basically. Up to now, a lot of lead-free perovskite materials have been developed, such as Bi-, Cu-, and Mn-based perovskites (Jiang et al., 2020b). Nevertheless, their material properties and corresponding device performance still cannot compete with the lead halide perovskites. To reduce the toxicity of perovskite materials, exploring novel lead-free perovskite materials should be further pursued. In this case, high-throughput computational screening and density functional theory can be combined to discover new perovskites with superior properties.
4) Doping is an effective way to modify perovskite polycrystalline thin films; however, doping perovskite NCs is relatively sluggish. Moreover, although doping in perovskite NCs have been demonstrated to improve emission properties, their application in LED devices should be explored. Guided by theoretical calculation, more rational, and effective doping will be raised to improve the properties of perovskite NCs and the EQE of LEDs.
5) In comparison to green, red, and yellow emission, blue-light perovskite NCs are relatively rare and the PLQY is smaller, leading to blue LEDs with low EQE. To overcome this point, effective B-site doping should be conducted in CsPbCl_3_ NCs, which may solve the bottleneck of blue-emission devices. Meanwhile, some B-site ions, such as Mn doping, can lead to broad emission due to existence of self-trapped excitons, which demonstrate the potential of applications in white-light applications.
